# The Impact of the COVID-19 Pandemic on Influenza Vaccination Coverage Among Young U.S. Children: A Socioeconomic Analysis

**DOI:** 10.3390/covid5020020

**Published:** 2025-02-10

**Authors:** Tawny Saleh, Mina Shirazi, Mary C. Cambou, Karin Nielsen-Saines

**Affiliations:** 1 Department of Medicine and Preventive Medicine, David Geffen School of Medicine at UCLA, Los Angeles, CA 90095, USA; 2 School of Electrical and Computer Engineering, University of Tehran, North Kargar Street, Tehran 1439957131, Iran; 3 Division of Infectious Diseases, Department of Medicine, David Geffen School of Medicine at UCLA, Los Angeles, CA 90095, USA; 4 Division of Pediatric Infectious Diseases, Department of Pediatrics, David Geffen School of Medicine at UCLA, Los Angeles, CA 90095, USA

**Keywords:** influenza vaccination, children, COVID-19 pandemic, socioeconomic disparities, public health strategies

## Abstract

The COVID-19 pandemic disrupted healthcare delivery across the United States (U.S.), including childhood vaccine administration. This study analyzed data from the National Health Interview Survey (NHIS), a nationally representative survey of the U.S. population, assessing trends and predictors of influenza vaccination uptake among children ≤ 5 years before and amid the COVID-19 pandemic. Influenza vaccination coverage declined significantly, from 56% in 2019 to 46% in 2022 (*p* < 0.001). Age-specific declines were notable, with rates dropping among one-year-olds from 68% to 53%, two-year-olds from 63% to 49%, and infants from 31% to 24% (*p* < 0.001). Logistic regression revealed African American children had lower odds of vaccination compared to non-Hispanic White children (OR = 0.70, *p* < 0.001), while Asian children had higher odds (OR = 1.32, *p* = 0.018). Uninsured children were less likely to be vaccinated than insured children (OR = 0.71, *p* = 0.022). Regional analysis showed the Northeast had the highest vaccination rates (60% in 2019, 56% in 2022), while the South had the lowest (52% in 2019, 41% in 2022). These findings underscore the need for targeted strategies to address socioeconomic disparities and improve influenza vaccine uptake in young children in the U.S.

## 1. Introduction

Influenza poses a significant public health challenge, contributing to considerable morbidity and mortality in the United States. Between the 2017–2018 and 2019–2020 influenza seasons, the virus was responsible for an estimated 29–41 million cases annually, resulting in as many as 52,000 deaths per year [1]. During the early phases of the COVID-19 pandemic, influenza activity sharply declined, due to widespread public health measures such as masking and school closures. However, by the 2021–22 season, influenza cases began returning to pre-pandemic levels, coinciding with the relaxation of these measures [1-3].

Young children, particularly those ≤5 years of age, remain at the highest risk for severe complications, with recent seasons recording elevated pediatric hospitalization rates [1-7]. Despite these challenges, influenza vaccination—the most effective defense against the virus—has shown persistently low coverage. For instance, during the 2021–22 season, only 53.3% of children aged 6 months to 17 years were vaccinated, continuing a trend of suboptimal uptake that existed prior to the pandemic [1,8-10]. Data from earlier seasons, such as 2019–20 and 2020–21, demonstrated the highest vaccination coverage in recent years, while subsequent seasons showed notable decreases, beginning in 2021–22 [11-13]. The COVID-19 pandemic introduced additional complexities, including altered healthcare-seeking behaviors, disruptions to routine immunization schedules, and heightened vaccine hesitancy among parents [11-16]. Preliminary studies during this period have reported significant reductions in vaccination coverage, emphasizing the urgent need to address barriers and ensure equitable vaccine access [12,13,16]. The burden of influenza is not evenly distributed among young children, with those from minority groups experiencing disproportionate impacts [8,9]. These disparities are compounded by consistently lower vaccination coverage among these children, further contributing to their higher risk of severe outcomes [8,9]. African American, Hispanic, and American Indian/Alaska Native children face disproportionately higher rates of severe influenza outcomes when compared to White children. Hospitalization rates are 1.9–3 times higher, ICU admissions 2–3.5 times higher, and in-hospital mortality rates 3–4.4 times higher, underscoring persistent systemic inequities [6,8]. Improved influenza vaccine coverage and early use of antiviral treatment may mitigate these disparities, potentially reducing severe outcomes among young children from minority groups [8,9].

While prior studies documented declines in vaccination rates during the COVID-19 pandemic [13], specific analyses focusing on influenza vaccination trends among U.S. children aged ≤ 5 years are lacking. The National Health Interview Survey (NHIS), a nationally representative source of health data, offers a unique opportunity to examine vaccination coverage in this vulnerable population [17,18]. To our knowledge, this study is the first to leverage NHIS public use microdata from 2019 and 2022 to assess influenza vaccination coverage trends before and during the pandemic, addressing a critical gap in understanding how socioeconomic factors influence vaccine uptake [17,18]. We hypothesize that the decline in vaccination coverage among children aged ≤ 5 years is associated with socioeconomic and demographic factors, including race, geographic region, family income, pediatric visit frequency, and parental education, which may have amplified existing health disparities during the COVID-19 pandemic. By identifying key socioeconomic predictors of vaccine uptake, this study provides evidence-based insights to inform public health strategies aimed at reducing disparities and improving vaccination rates among young children.

## 2. Methods

### 2.1. Data Source and Study Design

Data were obtained from the Integrated Public Use Microdata Series (IPUMS) adaptation of the National Health Interview Survey (NHIS) for the years 2019 and 2022 [17,18]. The NHIS, conducted by the National Center for Health Statistics, is a nationally representative survey of the U.S. civilian, non-institutionalized population, focusing on health behaviors, healthcare access, and health status. Utilizing a multistage sampling design, the NHIS ensures its findings are representative at the national level [17,18]. NHIS data collection methods in 2019 and 2022 were consistent, with similar survey designs, sampling methodologies, and core questionnaire structures, ensuring reliable comparisons of health data over time [17,18].

This study examines influenza vaccination coverage among children aged ≤ 5 years, comparing 2019, before the COVID-19 pandemic, to 2022, reflecting amid-pandemic vaccination behaviors during the 2022–2023 influenza season. Data from 2020 and 2021 were excluded due to methodological changes in the NHIS during the pandemic, including a shift to telephone surveys and lower response rates, which may introduce bias and compromise comparability with other years. By focusing on 2019 and 2022, this analysis provides a clearer assessment of shifts in influenza vaccination uptake among young U.S. children during the COVID-19 pandemic.

### 2.2. Measures

#### 2.2.1. Primary Outcome

The primary outcome of this study was the influenza vaccination status of children aged ≤ 5 years, determined from parent or guardian responses in the NHIS questionnaires [17,18]. Vaccination status was recorded as a binary variable, categorized as vaccinated (1) or not vaccinated (0). The structure of this survey question captured the seasonal influenza vaccine schedule, which is typically offered during the fall and winter in the United States. Influenza vaccine coverage was assessed based on an affirmative response to the survey question. This approach facilitated the evaluation of vaccination behaviors during the pre-COVID-19 pandemic (2019) and during the COVID-19 pandemic (2022) periods, offering insights into how vaccination coverage evolved over time.

#### 2.2.2. Independent Variables

Key independent variables included survey year, parental education, family income relative to the federal poverty level (FPL), region of residence, recency of the last doctor visit, and child’s age.

- The survey year was treated as a binary variable, representing 2019 (pre-COVID-19 pandemic) and 2022 (post-COVID-19 pandemic), to compare vaccination rates before and during the COVID-19 pandemic.
- Parental education was categorized into 11 levels, ranging from Grade 1–11, 12th grade with no diploma, GED or equivalent, high school graduate, some college with no degree, associate degree (occupational or academic program), bachelor’s degree, master’s degree, professional school degree (e.g., MD, JD), and doctoral degree (e.g., PhD, EdD). This detailed categorization provided granularity to evaluate how varying levels of educational attainment influenced vaccination behaviors.
- Family income was classified into three groups relative to the federal poverty level: less than 100% FPL, 100–199% FPL, and 200% or more FPL. These categories facilitated an exploration of socioeconomic disparities in influenza vaccination coverage.
- The recent doctor visit, an important healthcare utilization measure, was dichotomized into visits occurring within the past year versus more than a year ago.
- The child’s age was recorded in years. Notably, children aged 6–12 months fall within the initial eligibility window for receiving the first dose of the influenza vaccine in accordance with U.S. immunization guidelines [4].
- Region of residence was defined based on U.S. Census Bureau divisions, which included the following:
  1. **Northeast**: Maine, New Hampshire, Vermont, Massachusetts, Rhode Island, Connecticut, New York, New Jersey, and Pennsylvania.
  2. **Midwest**: Ohio, Indiana, Illinois, Michigan, Wisconsin, Minnesota, Iowa, Missouri, North Dakota, South Dakota, Nebraska, and Kansas.
  3. **South**: Delaware, Maryland, Virginia, West Virginia, North Carolina, South Carolina, Georgia, Florida, Kentucky, Tennessee, Alabama, Mississippi, Arkansas, Louisiana, Oklahoma, and Texas.
  4. **West**: Montana, Idaho, Wyoming, Colorado, New Mexico, Arizona, Utah, Nevada, Washington, Oregon, California, Alaska, and Hawaii.

For statistical analysis, children residing in the Northeast were designated as the reference group. This regional categorization enabled comparisons across distinct geographic areas to assess variations in vaccination behaviors.

#### 2.2.3. Control Variables

Control variables were included to adjust for potential confounding factors that might influence vaccination uptake. These included race and ethnicity, which were categorized separately. Race was defined as a classification based on physical traits and shared ancestry, including categories such as White, Black/African American, and Asian. Ethnicity, on the other hand, was defined as a cultural identity, with respondents classified as Hispanic or Non-Hispanic. These two variables were analyzed independently to avoid conflating racial and ethnic identities. Insurance status was also included as a control variable, dichotomized as insured (1) or uninsured (0). These control variables ensured that associations between independent variables and vaccination status were not confounded.

### 2.3. Statistical Analysis

Descriptive statistics summarized demographic and health-related characteristics for the 2019 and 2022 survey years. Bivariate analyses, including Chi-Square tests for categorical variables and Mann–Whitney U Tests for continuous variables, identified significant differences in vaccination coverage across the two periods, highlighting potential disparities before and after the COVID-19 pandemic. Logistic regression models were used to evaluate predictors of influenza vaccination, incorporating factors such as healthcare access, demographic variables, and socioeconomic characteristics. The framework followed the Andersen Behavioral Model [19,20], frequently applied in healthcare utilization studies, and was further informed by recent findings from Du et al., 2023 [21]. Model diagnostics included the Akaike Information Criterion (AIC), Bayesian Information Criterion (BIC), and the Area Under the Receiver Operating Characteristic (ROC) Curve to assess goodness-of-fit and predictive performance [22]. Sensitivity analyses were conducted to validate findings, ensuring consistency across models.

Subgroup analyses were conducted to explore disparities in influenza vaccination rates by key demographic and socioeconomic factors. Separate logistic regression models were constructed for each subgroup, focusing on the following:

- Parental Education: Vaccination rates were compared between children of parents with a bachelor’s degree or higher (highly educated) and those with less than a bachelor’s degree (non-highly educated).
- Region of Residence: Regional disparities were analyzed, comparing vaccination rates among children in the Northeast, Midwest, South, and West. The Northeast was designated as the reference group.
- Income Level: Family income categories (<100% FPL, 100–199% FPL, and ≥200% FPL) were analyzed to examine socioeconomic disparities.
- Race/Ethnicity: Vaccination rates among non-Hispanic White, African American, Hispanic, and Asian children were compared, with non-Hispanic White children as the reference group.

All statistical analyses were conducted using STATA version 18, with a significance level of α < 0.05 [23]. This approach addresses the complexities introduced by the COVID-19 pandemic and integrates demographic and healthcare access factors to identify vaccination disparities. The study used publicly available, de-identified data from the NHIS and was deemed exempt from the University of California, Los Angeles (UCLA) Institutional Review Board (IRB) oversight.

## 3. Results

An analysis of the NHIS data for 2019 (pre-COVID-19 pandemic) and 2022 (amid the COVID-19 pandemic) revealed significant trends in influenza vaccination among children aged ≤ 5 years of age. The 2019 data encompassed 2858 children, with a vaccination coverage estimate of 56% (*n* = 1601) (Table 1). In contrast, the 2022 data, which represented a larger cohort of 4778 children, had a vaccination coverage estimate of 46% (n = 2216), marking a significant decrease in vaccination rates over the observed period (*p* < 0.001) (Table 1). We observed a consistent gender distribution in both years, suggesting minimal influence of gender on vaccination status (Table 1).

### 3.1. Age-Specific Trends

Significant declines were noted in age-specific vaccination rates, particularly among one and two-year-olds, which dropped from 67% and 63% in 2019 to 53% and 49% in 2022, respectively (*p* < 0.001) (Table 1). This trend also extended to infants, whose vaccination rates fell from 31% in 2019 to 24% in 2022 (*p* < 0.001) (Table 1). Despite regular healthcare engagement, evidenced by children who had visited a doctor in the preceding year, influenza vaccination coverage decreased from 57% in 2019 to 47% in 2022 (*p* < 0.001) (Table 1), highlighting a general decline in vaccine uptake among these children.

### 3.2. Insurance and Regional Disparities

Although most children were insured in 2019 and 2022 (96% and 97%, respectively), insured children had significantly higher influenza vaccination coverage, with approximately 57% and 47% receiving the vaccine, respectively, as compared to uninsured children, who had lower estimates at 37% and 34% for the respective years. (*p* < 0.001) (Table 1). Moreover, regional disparities in influenza vaccination coverage were also evident. In 2019, the Northeast demonstrated the highest coverage at 60%, followed by the Midwest (59%) and the West (57%), while the South had the lowest coverage (51%) (*p* < 0.001; Table 1). By 2022, all regions experienced declines in influenza vaccine coverage. However, the Northeast remained the region with the highest influenza coverage at 56%, and the South exhibited the most pronounced decline to 41% (*p* < 0.001) (Table 1). While the Midwest and West also showed reductions in influenza vaccine coverage, these changes were not statistically significant compared to their 2019 levels (*p* > 0.05) (Table 1). Despite the overall decrease, the regional pattern of disparities in influenza vaccination coverage persisted over time (Table 1).

### 3.3. Socioeconomic and Ethnic Disparities

**Figure 1.**
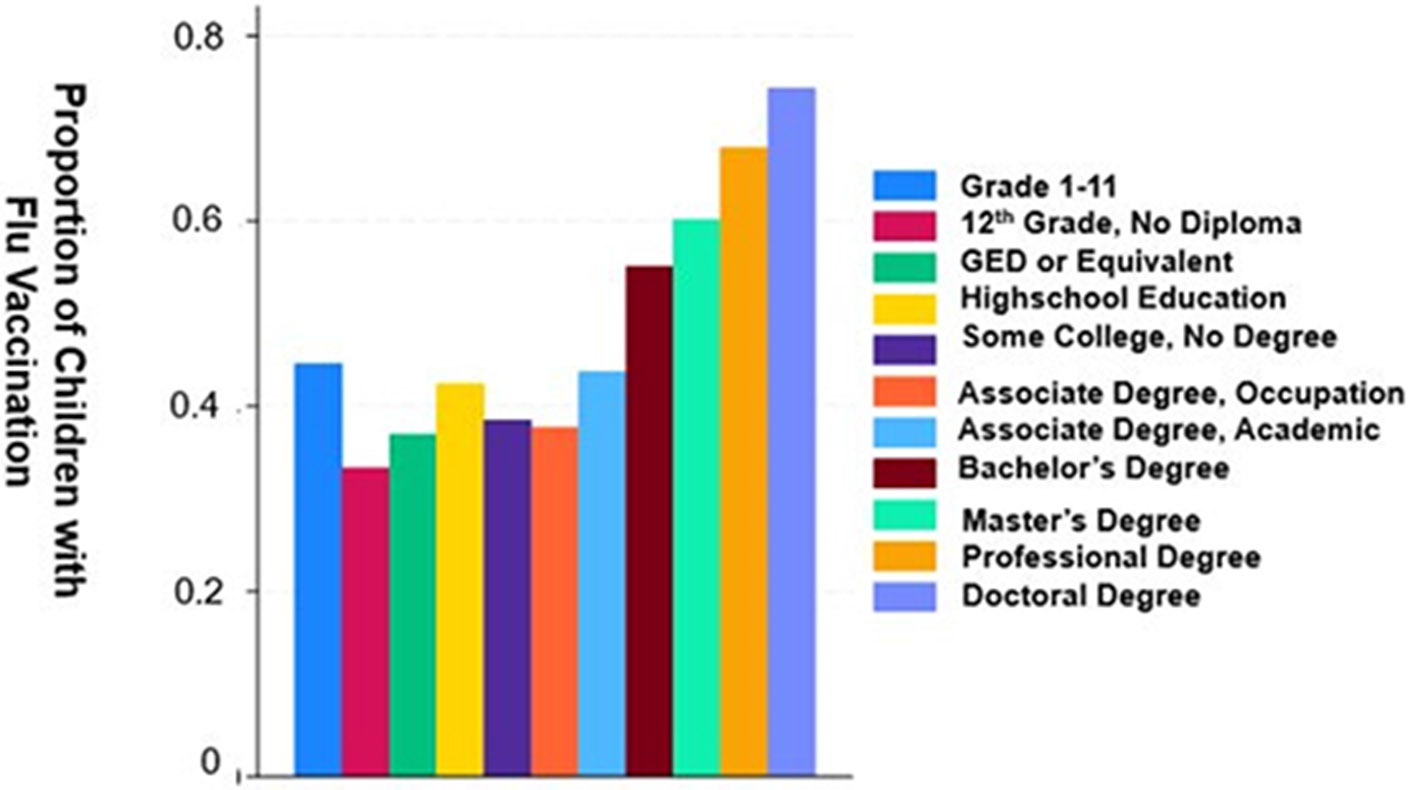
Influenza vaccination rates by educational attainment. Associations between the level of parental educational attainment and mean rates of influenza vaccination in children 5 years of age or less. Notably, parents with doctoral degrees had children with the highest mean vaccination rates, significantly outpacing those with lower educational levels. The mean vaccination rates appear to increase progressively with higher education levels, implying that educational attainment is likely a key determinant in health behavior related to the influenza vaccine. Statistical Values: The graph suggests that the mean vaccination rate for individuals with a Doctoral degree is approximately 0.8, indicating that, on average, 80% of individuals in this category received an influenza vaccine. In contrast, those who completed less than the 12th grade of high school (Grades 1–11) have a mean vaccination rate closer to 0.4, suggesting that around 40% in this group were vaccinated. These values demonstrate a doubling in vaccination rates between the lowest and highest education levels depicted.

**Figure 2.**
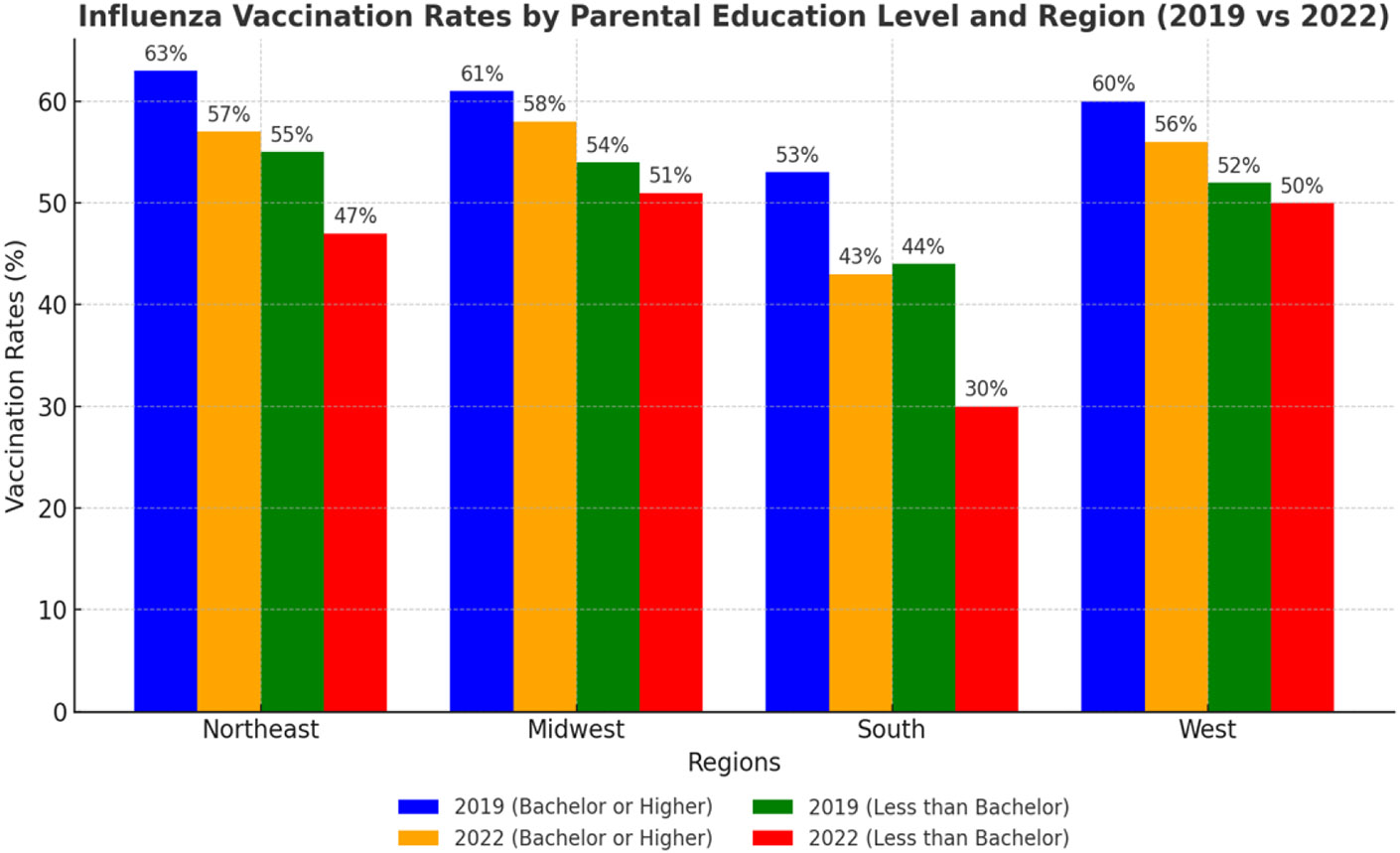
Influenza vaccination rates by parental education level and region (2019 vs. 2022). Influenza vaccination rates (%) among children aged ≤ 5 years across U.S. regions in 2019 and 2022, stratified by parental education level. Bars represent vaccination rates for children whose parents had either a bachelor’s degree or higher (blue and orange bars) or less than a bachelor’s degree (green and red bars). The Northeast consistently had the highest vaccination rates, while the South demonstrated the lowest rates. A significant decline in vaccination coverage was observed across most regions and education levels from 2019 to 2022.

Socioeconomic factors, particularly parental education and household income, were significantly associated with vaccination coverage estimates, with children from higher education and income backgrounds consistently showing higher uptake of immunization against influenza. Of note, Individuals with doctoral degrees have the highest average vaccination uptake of approximately 80% (Figures 1 and 2). Pre-COVID-19 pandemic vaccination rates were 61% for children of parents with a bachelor’s degree or higher, with a decline to 52% during the COVID-19 pandemic (Table 1). In contrast, children of parents with a high school education or less experienced lower influenza coverage estimates dropped further, from 51% pre-COVID-19 pandemic to 37% during the pandemic (Table 1). For income, influenza vaccination coverage estimates for the highest quartile above the family poverty level (FPL) fell from 69% to 60% before and during the COVID-19 pandemic, while vaccination coverage estimates in the lowest quartile below the FPL experienced a more drastic reduction from 48% to 27% before and during the COVID-19 pandemic (Table 1).

Ethnic disparities also markedly affected influenza vaccination coverage estimates among young children from 2019 to 2022. Asian children maintained the highest influenza vaccination coverage, slightly decreasing from 58% in 2019 to 57% in 2022 (*p* < 0.001) (Table 1). African American children, conversely, experienced a significant drop in influenza vaccination rates, from 47% to 33%, having the lowest coverage among racial/ethnic groups (*p* < 0.001). Hispanic children also saw a notable decline in influenza vaccination uptake, from 56% to 42% (*p* < 0.001) (Table 1).

### 3.4. Logistic Regression Analysis

The logistic regression analysis presented in Table 2 provides a detailed view of the predictors of influenza vaccination uptake among children in 2019 and 2022. There was a significant downturn in vaccination uptake during the COVID-19 pandemic (OR = 0.62, *p* < 0.001, Table 2). Each additional year of a child’s age increased the likelihood of receiving the influenza vaccine (OR = 1.18, *p* < 0.001, Table 2). Parental education was a strong predictor; children with parents holding professional degrees were significantly more likely to be vaccinated for influenza (OR = 1.84, *p* = 0.001, Table 2). Insurance status also played a crucial role, with uninsured children having lower influenza vaccination (OR = 0.71, *p* = 0.022, Table 2). Additionally, a recent doctor’s visit significantly increased the odds of influenza vaccination uptake (OR = 5.68, *p* = 0.001, Table 2).

Ethnic disparities were notable; Black/African American children were less likely to be vaccinated for influenza compared to their White Non-Hispanic counterparts (OR = 0.70, *p* < 0.001, Table 2). Conversely, Asian children had higher influenza vaccination uptake (OR = 1.32, *p* = 0.018, Table 2). Region of living also influenced vaccination rates, with children in the South being less likely to be vaccinated for influenza than those in the Northeast (OR = 0.70, *p* < 0.001, Table 2). Income levels showed significant effects, with children from the highest income quartile more likely to be vaccinated compared to those from the lowest quartile (OR = 1.93, *p* < 0.001, Table 2). These results underscore the numerous several factors influencing influenza vaccination rates among young children (Table 2).

### 3.5. Subgroup Analysis

Subgroup analyses were conducted to examine the influence of geographic and socioeconomic factors on influenza vaccination rates among children in 2019 and 2022 (Table 3). Parental education and geographic region emerged as significant predictors, with vaccination rates varying across subgroups. In the Northeast, children with parents holding a bachelor’s degree or higher had the highest vaccination rates, declining from 63% in 2019 to 57% in 2022 (OR = 0.76, *p* < 0.001). Children of parents with less than a bachelor’s degree in the Northeast also experienced a decline in vaccination rates, from 55% in 2019 to 47% in 2022 (OR = 0.74, *p* < 0.001).

In the South, significant declines were observed among all educational subgroups, reflecting compounded disparities. Children of parents with a bachelor’s degree or higher experienced a decrease in vaccination rates from 53% in 2019 to 43% in 2022 (OR = 0.68, *p* < 0.001). Those with parents holding less than a bachelor’s degree saw a more pronounced decline, from 44% in 2019 to 30% in 2022 (OR = 0.55, *p* < 0.001). The South consistently demonstrated the lowest vaccination rates across regions, highlighting persistent inequities.

In the Midwest, vaccination rates remained stable compared to other regions, with no significant changes observed. Children of parents with a bachelor’s degree or higher experienced a slight decline in vaccination rates, from 61% in 2019 to 58% in 2022 (OR = 0.91, *p* = 0.274). Similarly, children of parents with less than a bachelor’s degree showed a decline from 54% in 2019 to 51% in 2022 (OR = 0.91, *p* = 0.312). These non-significant changes suggest that vaccination trends in the Midwest were less affected by the COVID-19 pandemic.

In the West, vaccination rates showed marginal declines that were not statistically significant across educational subgroups. Children with parents holding a bachelor’s degree or higher saw rates decrease from 60% in 2019 to 56% in 2022 (OR = 0.87, *p* = 0.281). Those with parents holding less than a bachelor’s degree experienced a slight decrease in vaccination rates from 52% in 2019 to 50% in 2022 (OR = 0.94, *p* = 0.345). These findings indicate that vaccination trends in the West were stable across educational levels.

Differences in vaccination rates based on the timing of doctor visits, family income, race/ethnicity, and age-specific trends were not statistically significant in subgroup analyses. Specifically, timing of doctor visits showed no significant subgroup differences (*p* = 0.214), and interaction effects between family income and region or education were also not statistically significant (*p* = 0.091). Similarly, race/ethnicity interaction effects by income or region did not yield significant results (*p* = 0.127). Age-specific trends, although significant in the overall analysis, did not demonstrate significant interaction effects with other predictors (*p* = 0.078). These non-significant findings underscore the complexity of interactions between socioeconomic, geographic, and demographic factors in determining vaccination uptake, highlighting the need for further research to address barriers effectively.

## 4. Discussion

This study revealed a significant decline in influenza vaccination coverage among children ≤ 5 years, with rates dropping from 51% in 2019 to 41% in 2022. This decline reflects the compounded challenges of vaccine hesitancy exacerbated by the COVID-19 pandemic [24]. Regional disparities were evident, with the South experiencing the largest decline, from 51% in 2019 to 41% in 2022, compared to a smaller decline in the Northeast, where rates fell from 60% to 56%. These disparities underscore the critical need for region-specific public health interventions to address vaccine hesitancy and underlying socioeconomic barriers [25-27].

Socioeconomic factors, including parental education, income, and healthcare access, emerged as pivotal determinants of vaccination uptake. Expanding healthcare access and providing additional support to low-income families may reduce these disparities and improve vaccination rates [25-27]. Vaccine fatigue, characterized by reduced motivation for vaccination due to perceived burdens, burnout, or pandemic-related stress [28-30], may also explain part of the decline observed in 2022. This phenomenon emphasizes the importance of empathetic communication strategies to combat misinformation, rebuild trust, and address barriers to routine immunizations [28-30].

Both this study and the National Immunization Survey-Flu (NIS-Flu) identified declines in influenza vaccination rates during the COVID-19 pandemic. Our findings demonstrated a 10-percentage-point decline among children ≤ 5 years, while NIS-Flu data reported a 6.3-percentage-point decline among children aged 6 months–17 years [31]. Differences in reported values may stem from variations in methodology, as NHIS relies on parent-reported data, while NIS-Flu uses provider-reported data with more granular, state-level estimates [17,18,32]. These complementary sources provide valuable insights but highlight the need for future efforts to reconcile differences between datasets and validate national coverage estimates.

## 5. Limitations

This study has different limitations. First, its cross-sectional design limits the ability to infer causality. Reliance on parent-reported vaccination data introduces potential recall bias, particularly for earlier survey years. The larger sample size in 2022, implemented by NHIS to address pandemic-related response rate reductions, enhances the dataset’s representativeness but may also amplify observed differences between 2019 and 2022. Future studies should consider normalizing sample sizes across survey years to minimize potential bias.

The exclusion of 2020 and 2021 data due to methodological inconsistencies in NHIS limits the ability to analyze vaccination trends during the pandemic’s peak [23]. Additionally, immigration status, an important determinant of healthcare access and vaccination behavior [33], was not included in the dataset. Its absence may confound associations between insurance coverage and vaccination rates. Future research should integrate immigration status to better understand its impact on vaccination disparities. Finally, this study utilized logistic regression methods, which may not fully account for the hierarchical structure of data, such as regional or state-level clustering. Future studies should explore the use of multilevel modeling to capture these interactions [34], offering more granular insights into the structural and individual factors influencing vaccination behaviors.

## 6. Conclusions

This study highlights a significant decline in influenza vaccination rates among young children during the COVID-19 pandemic, driven by socioeconomic, regional, and racial disparities. These findings underscore the importance of targeted public health strategies, including region-specific educational outreach, policy interventions to improve healthcare access, and effective communication efforts to address vaccine hesitancy and vaccine fatigue. Future research should focus on integrating immigration status and employing multilevel analyses to better understand the structural and individual factors influencing vaccination rates. Validating vaccination trends across datasets, such as NHIS and NIS-Flu, will further inform interventions aimed at restoring influenza vaccination rates and reducing disparities.

## Figures and Tables

**Table 1.** Characteristics of the NHIS Survey population and influenza vaccination coverage among American children aged 0–5 Years in 2019 and 2022.

Variable	2019	2022	*p*-Value
**Total Children Surveyed**	2858	4778	-
**Vaccinated (%)**	56	46	<0.001
**Average Age (years)**	2.40 (±1.73)	2.49 (±1.78)	<0.001
**Male Proportion (%)**	50.5	50.9	0.12
**Insured Children (%)**	96.2	97.2	<0.001
**Uninsured Children (%)**	3.8	2.8	<0.001
**Vaccination Coverage of Insured (%)**	57	47	<0.001
**Vaccination Coverage of Uninsured (%)**	37	34	<0.001
**Had Doctor’s Visit in Past Year (%)**	92	89	0.02
**Vaccination Among Doctor Visit Subgroup (%)**	57	47	<0.001
**Vaccination by Region: Northeast (%)**	60	56	<0.001
**Vaccination by Region: Midwest (%)**	59	46	0.09
**Vaccination by Region: West (%)**	57	49	0.12
**Vaccination by Region: South (%)**	50	41	<0.001
**Parental Education: Bachelor’s or Higher (%)**	61	51	<0.001
**Parental Education: High School or Less (%)**	51	37	<0.001
**Income: Highest Quartile (%)**	69	60	<0.001
**Income: Lowest Quartile (%)**	48	27	<0.001
**Non-Hispanic Asian Vaccination Coverage (%)**	58	57	<0.001
**African American Vaccination Coverage (%)**	47	33	<0.001
**Hispanic Vaccination Coverage (%)**	56	42	<0.001
**Age Group (years)**			
0	31	24	<0.001
1	67	53	<0.001
2	63	49	<0.001
3	60	53	<0.001
4	60	52	<0.001
5	57	48	<0.001

**Table 2.** Factors associated with influenza vaccination odds among young children in the United States, 2019 and 2022.

Variable	Odds Ratio (OR)	95% CI	*p*-Value
**Survey Year (2022 vs. 2019)**	0.62	0.63–0.67	<0.001
**Age (per year increase)**	1.18	1.14–1.20	<0.001
**Ethnicity (NH Black vs. NH White)**	0.70	0.61–0.80	<0.001
**Ethnicity (NH Asian vs. NH White)**	1.32	1.05–1.65	0.018
**Region (South vs. Northeast)**	0.70	0.66–0.75	<0.001
**Parental Education (Professional Degree)**	1.84	1.30–2.60	0.001
**Income Quartile (Highest vs. Lowest)**	1.93	1.46–2.55	<0.001
**Insurance Status (Uninsured)**	0.71	0.56–0.91	0.022
**Recent Doctor’s Visit (Yes)**	5.68	2.09–15.44	0.001

Odds ratios (OR) represent the likelihood of being vaccinated against influenza. OR values greater than 1.0 indicate higher odds of vaccination, while OR values less than 1.0 indicate lower odds of vaccination. For example, children with a recent doctor’s visit (OR = 5.68) had significantly higher odds of being vaccinated, while children living in the South (OR = 0.70) had lower odds compared to children in the Northeast. NH: Non-Hispanic.

**Table 3.** Odds Ratios for Influenza Vaccination in 2022 Compared to 2019 by Geographic Region and Parental Education Level. This table displays the odds ratios (OR), 95% confidence intervals (CI), and *p*-values for influenza vaccination rates in 2022 relative to 2019 among children grouped by geographic region (Northeast and South) and parental education level (Bachelor’s Degree or Higher, Less than a Bachelor’s Degree). Odds ratios less than 1 indicate a decline in vaccination rates in 2022 compared to 2019, with statistically significant results (*p* < 0.001) highlighted.

Subgroup	2019 Vaccination Rate (%)	2022 Vaccination Rate (%)	Odds Ratio	95% CI	*p*-Value
**Northeast**	63	57	0.76	0.70–0.83	<0.001
**South**	53	43	0.68	0.62–0.74	<0.001
**Parental Education Bachelor’s Degree or Higher**	60	52	0.73	0.68–0.78	<0.001
**Parental Education Less than a Bachelor’s degree**	47	35	0.59	0.53–0.66	<0.001

## Data Availability

Data for this study is available upon reasonable request. As this study utilized publicly available datasets, no new data were created.
